# Supplementary material for: Evaluating the Relative Vaccine Effectiveness of Adjuvanted Trivalent Influenza Vaccine Compared to High-Dose Trivalent and Other Egg-Based Influenza Vaccines among Older Adults in the US during the 2017–2018 Influenza Season
Source: Vaccines (Basel). 2020 Aug 7;8(3):446. doi: 10.3390/vaccines8030446 (PMC7563546; doi:10.3390/vaccines8030446)
Supplement: Supplementary file 1 [file vaccines-08-00446-s001.pdf]

Supplementary Table S1. Baseline Demographic Characteristics – Unadjusted

Vaccine Cohort / Characteristic	aTIV N=234,313 A	TIV-HD N=1,269,855 B	QIVe-SD N=212,287 C	TIVe-SD N=106,491 D	SMD[1] A x B	SMD[1] A x C	SMD[1] A x D
**Mean age**	75.1	75.0	74.0	74.5	-0.02	**-0.19**	**-0.10**
SD	6.3	6.3	6.7	6.6			
Median	74	74	73	74			
**Age group (%)**							
65-74 years	50.2%	50.7%	56.7%	53.4%	0.01	**0.13**	0.07
75-84 years	34.1%	33.9%	29.1%	31.2%	0.00	**-0.11**	-0.06
≥85 years	15.8%	15.3%	14.3%	15.3%	-0.01	-0.04	-0.01
**Female (%)**	60.0%	59.9%	59.8%	60.5%	0.00	0.00	0.01
**Geographic region (%)**							
Northeast	15.8%	17.1%	15.5%	12.3%	0.04	-0.01	**-0.10**
Midwest	11.8%	18.9%	17.8%	9.9%	**0.20**	**0.17**	-0.06
South	53.4%	43.2%	39.7%	43.3%	**-0.20**	**-0.28**	**-0.20**
West	19.1%	20.8%	27.1%	34.4%	0.04	**0.19**	**0.35**
**Payer type (%)**							
Cash	0.5%	0.3%	0.8%	0.8%	-0.02	0.05	0.05
Medicaid	0.0%	0.1%	0.2%	0.1%	0.01	0.04	0.03
Medicare Part D	33.5%	28.8%	15.4%	15.6%	**-0.10**	**-0.43**	**-0.43**
Medicare	44.6%	36.4%	34.3%	37.7%	**-0.17**	**-0.21**	**-0.14**
Third party	21.3%	34.1%	48.6%	44.7%	**0.29**	**0.60**	**0.51**
Other/Unknown	0.1%	0.3%	0.6%	1.1%	0.03	0.08	0.12

1 SMD (absolute) ≥0.1, bolded in the table, indicates significance
SMD = standardized mean difference

Supplementary Table S2. Baseline Clinical Characteristics – Unadjusted

Vaccine Cohort<br>Characteristic	aTIV<br>N=234,313<br>A	TIV-HD<br>N=1,269,855<br>B	QIVe-SD<br>N=212,287<br>C	TIVe-SD<br>N=106,491<br>D	SMD[1]<br>A x B	 A x C	 A x D
**Month of flu vaccination (%)**							
August	8.3%	5.6%	3.8%	1.9%	**-0.11**	**-0.19**	**-0.29**
September	27.2%	31.6%	26.8%	22.0%	**0.10**	-0.01	**-0.12**
October	40.4%	41.2%	41.3%	43.1%	0.02	0.02	0.05
November	13.9%	14.0%	16.6%	18.9%	0.00	0.07	**0.14**
December	5.3%	4.4%	6.3%	7.1%	-0.04	0.05	0.08
January	4.8%	3.1%	5.3%	6.9%	-0.09	0.02	0.09
**CCI score (%)**							
0	57.2%	55.1%	49.6%	45.3%	-0.04	**-0.15**	**-0.24**
1	20.7%	21.4%	22.3%	22.9%	0.02	0.04	0.05
2	11.8%	12.3%	13.8%	15.0%	0.01	0.06	0.09
3+	10.3%	11.3%	14.2%	16.9%	0.03	**0.12**	**0.19**
**Mean CCI score**	0.8	0.9	1.1	1.2	0.05	**0.17**	**0.27**
SD	1.3	1.3	1.5	1.5			
Median	0	0	1	1			
**Pre-index comorbidities (%)**							
Asthma	3.5%	3.8%	4.3%	4.7%	0.01	0.04	0.06
Blood disorders	0.3%	0.3%	0.3%	0.3%	0.00	0.00	0.01
Chronic lung disease	8.3%	8.8%	10.2%	11.4%	0.02	0.07	0.11
Diabetes	19.8%	21.5%	26.1%	29.5%	0.04	**0.15**	**0.23**
Heart disease	11.9%	12.5%	14.4%	16.2%	0.02	0.07	**0.12**
Kidney disorders	8.2%	8.7%	10.0%	11.9%	0.02	0.07	**0.13**
Liver disorders	2.1%	2.1%	2.8%	3.1%	0.00	0.04	0.06
Neurological or neurodevelopmental conditions	4.7%	4.9%	5.2%	5.3%	0.01	0.02	0.03
Weakened immune system[2]	9.6%	9.9%	9.9%	9.8%	0.01	0.01	0.00
IBD	0.6%	0.6%	0.6%	0.6%	0.00	0.00	0.00

Vaccine Cohort<br>Characteristic	aTIV<br>N=234,313<br>A	TIV-HD<br>N=1,269,855<br>B	QIVe-SD<br>N=212,287<br>C	TIVe-SD<br>N=106,491<br>D	SMD[1]<br>A x B	 A x C	 A x D
**Indicators of frail health status (%)**							
Home oxygen use	3.9%	4.1%	4.7%	5.1%	0.01	0.04	0.06
Wheelchair use	2.1%	2.3%	2.8%	2.8%	0.01	0.05	0.05
Walker use	3.2%	3.3%	3.6%	3.7%	0.01	0.02	0.03
Dementia	1.3%	1.3%	1.3%	1.5%	0.00	0.00	0.02
Urinary catheter use	0.4%	0.4%	0.4%	0.4%	0.00	0.00	0.00
Falls	0.9%	0.9%	0.8%	0.9%	0.00	0.00	0.01
Fractures	0.6%	0.6%	0.6%	0.6%	0.00	0.00	0.01
**Pre-index hospitalization (%)**	7.7%	7.9%	8.2%	8.6%	0.01	0.02	0.03
**Mean pre-index outpatient pharmacy costs**	$2,400	$2,425	$2,380	$2,540	0.02	-0.01	0.07
SD	$6,037	$6,037	$6,094	$5,229			
Median	$861	$885	$807	$990			
**Mean inpatient costs**	$810	$826	$933	$1,064	0.01	0.02	0.03
SD	$9,183	$7,795	$10,924	$9,239			
Median	$0	$0	$0	$0			
**Mean outpatient medical costs**	$1,738	$1,692	$1,686	$1,734	0.00	-0.03	-0.03
SD	$6,309	$6,620	$6,571	$6,282			
Median	$400	$396	$380	$378			
**Mean TOTAL pre-index costs[3]**	$4,949	$4,943	$4,999	$5,338	0.00	-0.03	0.04
SD	$13,376	$12,274	$14,748	$13,109			
Median	$2,039	$2,043	$1,909	$2,140			

1 SMD (absolute) ≥0.1, bolded in the table, indicates significance

2 Including: HIV/AIDS; metastatic cancer and acute leukemia; lung or upper digestive or other severe cancer; lymphatic, head, neck, brain, or major cancer; breast, prostate, colorectal, or other cancer; and disorders of immunity

CCI = Charlson Comorbidity Index Score; IBD = Inflammatory bowel diseases (ulcerative colitis and Crohn's disease); SMD = Standardized mean difference

3 TOTAL = outpatient pharmacy + inpatient + outpatient medical
